# Identifying heat thresholds for South Africa towards the development of a heat-health warning system

**DOI:** 10.1007/s00484-023-02596-z

**Published:** 2023-12-29

**Authors:** Thandi Kapwata, Nada Abdelatif, Noah Scovronick, Michael T. Gebreslasie, Fiorella Acquaotta, Caradee Y. Wright

**Affiliations:** 1 Environment and Health Research Unit, South African Medical Research Council, Johannesburg, 2028 South Africa; 2 Department of Geography, Geoinformatics and Meteorology, University of Pretoria, Pretoria, 0028 South Africa; 3 Biostatistics Research Unit, South African Medical Research Council, Durban, 4001 South Africa; 4 Gangarosa Department of Environmental Health, Rollins School of Public Health, Emory University, Atlanta, GA 30322 USA; 5 School of Agriculture, Earth, and Environmental Sciences, University of KwaZulu-Natal, Durban, 3629 South Africa; 6 Department of Earth Science, University of Turin, Turin, Italy; 7 Environment and Health Research Unit, South African Medical Research Council, Pretoria, 0084 South Africa

**Keywords:** Heat-health warning systems, Mortality, Temperature metric, Thresholds

## Abstract

Exposure to heatwaves may result in adverse human health impacts. Heat alerts in South Africa are currently based on defined temperature-fixed threshold values for large towns and cities. However, heat-health warning systems (HHWS) should incorporate metrics that have been shown to be effective predictors of negative heat-related health outcomes. This study contributes to the development of a HHWS for South Africa that can potentially minimize heat-related mortality. Distributed lag nonlinear models (DLNM) were used to assess the association between maximum and minimum temperature and diurnal temperature range (DTR) and population-adjusted mortality during summer months, and the effects were presented as incidence rate ratios (IRR). District-level thresholds for the best predictor from these three metrics were estimated with threshold regression. The mortality dataset contained records of daily registered deaths (*n* = 8,476,532) from 1997 to 2013 and data for the temperature indices were for the same period. Maximum temperature appeared to be the most statistically significant predictor of all-cause mortality with strong associations observed in 40 out of 52 districts. Maximum temperature was associated with increased risk of mortality in all but three of the districts. Our results also found that heat-related mortality was influenced by regional climate because the spatial distribution of the thresholds varied according to the climate zones across the country. On average, districts located in the hot, arid interior provinces of the Northern Cape and North West experienced some of the highest thresholds compared to districts located in temperate interior or coastal provinces. As the effects of climate change become more significant, population exposure to heat is increasing. Therefore, evidence-based HHWS are required to reduce heat-related mortality and morbidity. The exceedance of the maximum temperature thresholds provided in this study could be used to issue heat alerts as part of effective heat health action plans.

**Supplementary Information:**

The online version contains supplementary material available at 10.1007/s00484-023-02596-z.

## Introduction

Climate change is projected to intensify the adverse health impacts of extreme heat by increasing their frequency, severity, and duration of heatwave events (Ebi et al. 2021; Zhao et al. 2022). Several record-breaking heatwaves have been reported in many parts of the world. In the summer of 2003, for example, Europe experienced its hottest heatwave in 500 years; studies showed that heat-related excess deaths during the 2003 European summer exceeded 70,000 (Luterbacher et al. 2004; Trigo et al. 2009; Christoph and Gerd 2004; Robine et al. 2008; Fouillet et al. 2006). Similarly, in 2010, Russia experienced its worst heatwave since records began that resulted in an estimated 55,000 deaths (Hoag 2014; Grumm 2011). In 2006 in the USA, California (which was the most affected state) recorded 655 heat-related deaths over 2 weeks (Coumou and Rahmstorf 2012; Knowlton et al. 2012, 2008). Devasting heatwaves which were associated with high numbers of deaths and illnesses have also been reported in Australia and India (Nitschke et al. 2011; Azhar et al. 2014; Mazdiyasni et al. 2017).

It is evident that research on health-related impacts of heatwaves is unevenly distributed as the majority of studies are concentrated in mid-latitude temperate regions including North America, Europe, eastern China, and Australia while lacking in lower middle-income settings such as Africa and South America (Campbell et al. 2018). For example, South Africa has experienced prolonged, intense heatwaves in recent years; however, there are no published articles that present the health effects of these events leading to an underestimation of the risks. Most reports about health outcomes attributed to extreme heat in the country are from media sources, such as recent media releases that revealed eight people, most of them farm workers, died from heatstroke in the Northern Cape province of the country during a heatwave in late January 2023 (AfricaNews 2023).

Given the severity of the impacts of extreme heat events, several countries (mainly in Europe and North America) have implemented heat-health warning systems (HHWS) to reduce heat-related morbidity and mortality (Casanueva et al. 2019; Kotharkar and Ghosh 2022). According to recommended methodologies, the development of HHWS should be based on knowledge of cause-effect relationships between temperature and the health of a given population (Kim et al. 2006; Montero et al. 2010). This information can then be used to estimate thresholds to trigger heat warning alerts (McGregor et al. 2015). However, the South African Weather Service (SAWS) currently issues heat warnings using city/town-specific absolute threshold values that are not associated with a negative human response (SAWS 2017).

Although recent South African studies have found strong associations between hot days and mortality especially in children and older adults (Wichmann 2017; Scovronick et al. 2018), these analyses were not restricted to summer months to model heat effects only. Furthermore, the methods used in these studies did not account for population size, which is an important confounder considering that previous studies show heat extremes often have substantially higher impacts when they occur in highly populated areas (Chebana et al. 2013; Harrington and Otto 2018).

Stakeholder participation at local level is necessary to ensure sustainability and effectiveness of HHWS (Climate-ADAPT 2023). In South Africa, there are three tiers of government, namely, national, provincial, and local. Local government comprises 52 districts nationally, and this administrative level is responsible for coordinating development and service delivery. The South African Heat Health Guidelines acknowledge that district municipalities play a crucial role in reducing the burden of disease due to heat exposure (Department of Health 2020). Therefore, districts should be incorporated in the design and implementation heat health action plans such as HHWSs.

Here, we used distributed lag nonlinear models (DLNM) using quasi-Poisson regression models to identify the most statistically significant temperature metric between maximum and minimum temperature and diurnal temperature range (DTR) while adjusting for population in each of the 52 districts in South Africa. We further estimated district-level heat thresholds for the most statistically significant temperature metric. This study enables recommendations for an appropriate exposure metric and the associated location-specific thresholds to issue heat alerts to contribute towards the development of a health-outcome evidenced HHWS for South Africa.

## Methods

### Mortality and population data

Statistics South Africa provided the mortality data for 1997–2013, which contains all deaths registered and collated through the South African civil registration system maintained by the Department of Home Affairs. A data quality assessment conducted by Statistics South Africa estimated that adult (15 years and older) death registrations were ~ 89% complete early on in the study period, increasing to ~ 94% completion by 2013, while the child death records had not been sufficiently reported (Stats SA 2014; Scovronick et al. 2018). Records with missing or incomplete information about location of death (district) and/or date of death were excluded from the dataset used for analysis. This study used all-cause mortality similar to most epidemiological studies because temperature-related mortality is often misclassified and underestimated (Hajat and Kosatky 2010; Alahmad et al. 2019). District-level population estimates for the study period were also obtained from Statistics South Africa.

### Temperature data

Data for maximum and minimum temperature were provided by the National Oceanographic and Atmospheric Administration (NOAA) and South Africa’s Agricultural Research Council (ARC). This dataset comprised daily minimum and maximum temperatures for each of the country’s 52 district municipalities. The NOAA dataset comprised daily data covering 30 district municipalities, while the ARC dataset covered 50 districts except the City of Johannesburg and Nelson Mandela Bay. Using the most complete datasets from the two sources, the final dataset consisted of temperature data from ARC for 34 district municipalities and NOAA data from 18 district municipalities. In-depth quality control was conducted on the dataset to exclude invalid values resulting from instrument error or human error during data uploading and capturing. The collation, quality control procedures, and processing of the temperature dataset are described in more detail in Scovronick et al. (2018). Daily DTR was calculated as the difference between maximum and minimum temperature.

Heat-related mortality studies often limit temperature data to summer months because this excludes the effects of cold temperature (Li et al. 2012; Basu 2009). Therefore, this study limits its temperature data from October to March, which are considered “summer” months in South Africa due to the warmer temperatures compared to the rest of the year.

### Statistical analysis

To explore the association between maximum temperature and minimum temperature and DTR on all-cause mortality, a distributed lag nonlinear model (DLNM) using a quasi-Poisson regression model was implemented to express the nonlinear exposure-lag-response relationship. This was performed separately for each district due to heterogeneity between the locations. The model was adjusted to account for the population of each district, and results are reported as IRR (incidence risk ratio) per 10,000 people. The R package “dlnm” was used to carry out the DLNM analysis (Gasparrini 2011).

To determine the threshold values for the significant temperature metrics for each district, threshold regression was used. Threshold regression models are a class of models where the predictors are believed to impact the outcome at different change points or thresholds. Threshold regression extends linear regression to allow coefficients to vary across different regions (Fong et al. 2017). These regions are identified as either being above or below a threshold value. The threshold parameter can be thought of as a change point, and the model provides an easy to interpret method of describing nonlinear relationships between an outcome and predictors (Fong et al. 2017). A model is fitted to obtain an estimate of the threshold and the coefficients on either side of it. The two regions are defined by a threshold value *γ* with (Gonzalo and Pitarakis 2002)

$${y}_{t}={x}_{t}\beta +{z}_{t}{\delta }_{1}+{\varepsilon }_{t} {\text{if}} -\infty <{\omega }_{t}\le \gamma$$

$${y}_{t}={x}_{t}\beta +{z}_{t}{\delta }_{2}+{\varepsilon }_{t} {\text{if}} \gamma <{\omega }_{t}<\infty$$

where ${y}_{t}$ is the dependent variable, ${x}_{t}$ is a $1\times {\text{k}}$ vector of covariates, $\beta$ is a $k\times 1$ vector of region-invariant parameters, ${\varepsilon }_{t}$ is an IID error with mean 0 and variance ${\sigma }^{2}$, ${z}_{t}$ is a vector of exogenous variables with region-specific coefficient vectors ${\delta }_{1}$ and ${\delta }_{2}$, and ${\omega }_{t}$ is a threshold variable that may be one of the variables in ${x}_{t}$ or ${z}_{t}$. The conditional least squares function is used to estimate the parameters of the threshold regression model for each district. Stata version 15.1 was used for the threshold regression analysis (StataCorp 2017).

## Results

### Descriptive statistics

The descriptive statistics of mortality and temperature metrics are illustrated in Supplementary Tables S1. Total warm-season mortality in South Africa from 1997 to 2013 was 4,066,276. As illustrated in Table S1, the eThekwini district (number 19) had the highest number of reported deaths for all causes (*n* = 284,598), and the Central Karoo district (number 8) recorded the lowest number of deaths (*n* = 6547). Both the highest maximum and lowest minimum temperatures of 47.3 and 0.84 °C, respectively, were observed in districts of KwaZulu-Natal province across all study years.

### DLNM

Maximum temperature was the most statistically significant temperature metric in 40 (77%) districts out 52 (Table 1). A majority of the districts showed a significant association (*p* < 0.05) with maximum temperature. Minimum temperature was second (7 districts, 13%) followed by DTR (3 districts, 6%). Maximum temperature was associated with increased risk of mortality per 10,000 people ranging from 3 to 164% (IRR = 1.03, coefficient = 0.03, 95% CI =  − 0.93–0.98, not statistically significant and IRR = 2.64, coefficient = 0.97, 95% CI =  − 31.69–33.63, not statistically significant), respectively. However, three district municipalities, namely, Buffalo City (Eastern Cape), Cape Winelands (Western Cape), and Ekurhuleni (Gauteng), showed that max temperature led to a reduction of 26%, 5%, and 6% in risk of mortality per 10,000 people, respectively.

**Table 1:** DLNM model results showing the effects of summer temperatures on mortality expressed as the incident rate ratio (IRR) per 10,000 people

District	Index	Coefficient	95% CI		IRR	*p* value
alfn	DTR	− 0.28	− 0.79	0.23	0.76	
	Max temp	0.26	0.10	0.41	1.29	***
	Min temp	0.29	0.00	0.58	1.34	**
amjb	DTR	− 0.14	− 0.61	0.33	0.87	
	Max temp	0.22	0.06	0.37	1.24	***
	Min temp	− 0.01	− 0.27	0.26	0.99	
amth	DTR	− 0.03	− 0.54	0.49	0.97	
	Max temp	0.41	0.27	0.54	1.50	***
	Min temp	0.14	− 0.09	0.37	1.15	
bffc	DTR	0.23	− 0.88	1.34	1.26	
	Max temp	− 0.30	− 0.73	0.13	0.74	
	Min temp	− 0.32	− 0.78	0.13	0.73	
bjnl	DTR	− 0.02	− 0.37	0.33	0.98	
	Max temp	0.64	0.47	0.80	1.89	***
	Min temp	0.25	0.08	0.42	1.29	***
cacd	DTR	− 0.23	− 0.73	0.26	0.79	
	Max temp	0.31	0.17	0.45	1.37	***
	Min temp	0.17	− 0.08	0.41	1.18	
chrh	DTR	0.12	− 0.40	0.64	1.13	
	Max temp	0.22	0.02	0.41	1.24	**
	Min temp	0.01	− 0.25	0.27	1.01	
cntk	DTR	0.24	− 0.93	1.40	1.27	
	Max temp	0.07	− 0.24	0.38	1.07	
	Min temp	− 0.57	− 1.07	− 0.07	0.57	**
coct	DTR	0.17	0.03	0.31	1.18	**
	Max temp	0.22	0.16	0.29	1.25	***
	Min temp	0.02	− 0.06	0.10	1.02	
cprc	DTR	− 0.07	− 0.41	0.27	0.94	
	Max temp	0.35	0.21	0.49	1.42	***
	Min temp	0.10	− 0.06	0.26	1.11	
cpwn	DTR	− 0.68	− 6.47	5.12	0.51	
	Max temp	− 0.05	− 2.49	2.39	0.95	
	Min temp	− 0.05	− 3.82	3.71	0.95	
ctoj	DTR	− 2.28	− 48.91	44.36	0.10	
	Max temp	0.97	− 31.69	33.63	2.64	
	Min temp	0.90	− 27.48	29.29	2.46	
ctot	DTR	0.401	− 3.76	4.57	1.49	
	Max temp	0.864	− 1.77	3.50	2.37	
	Min temp	0.26	− 2.33	2.85	1.30	
drkk	DTR	− 0.18	− 0.73	0.36	0.83	
	Max temp	0.29	0.03	0.54	1.33	**
	Min temp	− 0.10	− 0.31	0.10	0.90	
drsm	DTR	0.07	− 0.36	0.51	1.08	
	Max temp	0.49	0.30	0.69	1.64	***
	Min temp	0.04	− 0.11	0.19	1.04	
eden	DTR	0.19	− 0.31	0.69	1.21	
	Max temp	0.24	0.08	0.39	1.26	***
	Min temp	0.10	− 0.15	0.36	1.11	
ehln	DTR	0.11	− 0.33	0.56	1.12	
	Max temp	0.22	0.05	0.39	1.24	***
	Min temp	− 0.09	− 0.32	0.14	0.92	
ekrh	DTR	0.36	− 2.79	3.51	1.43	
	Max temp	− 0.06	− 2.09	1.97	0.94	
	Min temp	− 0.19	− 2.10	1.73	0.83	
ethk	DTR	− 0.26	− 1.68	1.16	0.77	
	Max temp	0.03	− 0.93	0.98	1.03	
	Min temp	− 0.08	− 0.89	0.73	0.92	
frnb	DTR	0.12	− 0.33	0.56	1.12	
	Max temp	0.26	0.08	0.44	1.29	***
	Min temp	− 0.05	− 0.22	0.11	0.95	
fzld	DTR	− 0.03	− 0.45	0.39	0.97	
	Max temp	0.26	0.07	0.45	1.30	***
	Min temp	0.09	− 0.12	0.30	1.10	
grtb	DTR	− 0.39	− 1.00	0.23	0.68	
	Max temp	0.24	0.06	0.42	1.27	**
	Min temp	0.09	− 0.19	0.37	1.09	
grts	DTR	− 0.30	− 0.55	− 0.06	0.74	**
	Max temp	0.39	0.24	0.54	1.48	***
	Min temp	0.11	− 0.05	0.27	1.12	
ilmb	DTR	− 0.43	− 0.89	0.02	0.65	*
	Max temp	0.60	0.41	0.79	1.82	***
	Min temp	− 0.15	− 0.42	0.13	0.86	
jgqb	DTR	− 0.09	− 0.65	0.47	0.91	
	Max temp	0.12	− 0.09	0.32	1.12	
	Min temp	0.18	− 0.03	0.39	1.20	*
jhtg	DTR	− 0.12	− 0.86	0.61	0.89	
	Max temp	0.26	− 0.09	0.61	1.30	
	Min temp	− 0.16	− 0.52	0.20	0.85	
ljwl	DTR	− 0.25	− 0.71	0.21	0.78	
	Max temp	0.43	0.26	0.60	1.54	***
	Min temp	0.17	0.00	0.34	1.19	**
mngn	DTR	0.13	− 0.33	0.59	1.14	
	Max temp	0.27	0.07	0.46	1.30	***
	Min temp	0.01	− 0.14	0.17	1.01	
mopn	DTR	0.05	− 0.23	0.32	1.05	
	Max temp	0.42	0.31	0.53	1.52	***
	Min temp	0.14	− 0.04	0.32	1.15	
ngmm	DTR	0.02	− 0.29	0.32	1.02	
	Max temp	0.40	0.25	0.56	1.49	***
	Min temp	0.06	− 0.05	0.16	1.06	
nkng	DTR	0.07	− 0.28	0.42	1.07	
	Max temp	0.28	0.16	0.41	1.33	***
	Min temp	0.02	− 0.15	0.18	1.02	
nlmb	DTR	0.10	− 0.30	0.51	1.11	
	Max temp	0.27	0.11	0.44	1.31	***
	Min temp	0.00	− 0.13	0.13	1.00	
nmkw	DTR	0.80	− 0.80	2.40	2.22	
	Max temp	0.36	− 0.37	1.09	1.43	
	Min temp	0.19	− 0.43	0.81	1.21	
ortm	DTR	− 0.03	− 0.66	0.60	0.97	
	Max temp	0.26	0.08	0.44	1.29	***
	Min temp	0.30	0.00	0.60	1.35	*
ovrb	DTR	− 0.04	− 0.77	0.69	0.96	
	Max temp	0.18	− 0.05	0.40	1.19	
	Min temp	0.07	− 0.14	0.27	1.07	
pxks	DTR	− 0.10	− 0.56	0.35	0.90	
	Max temp	0.54	0.25	0.83	1.71	***
	Min temp	0.17	− 0.08	0.42	1.19	
sdbn	DTR	0.22	− 0.20	0.64	1.25	
	Max temp	0.31	0.12	0.50	1.37	***
	Min temp	− 0.17	− 0.37	0.03	0.85	*
ssnk	DTR	− 0.06	− 0.61	0.50	0.95	
	Max temp	0.41	0.24	0.58	1.51	***
	Min temp	0.34	0.06	0.62	1.40	**
synd	DTR	0.42	− 0.14	0.98	1.52	
	Max temp	0.45	0.20	0.70	1.56	***
	Min temp	0.10	− 0.17	0.36	1.10	
thbm	DTR	− 0.32	− 0.71	0.08	0.73	
	Max temp	0.22	0.02	0.41	1.24	**
	Min temp	− 0.03	− 0.20	0.14	0.97	
uguc	DTR	− 0.31	− 0.68	0.05	0.73	*
	Max temp	0.12	− 0.09	0.33	1.13	
	Min temp	0.29	0.11	0.47	1.33	***
umgn	DTR	0.48	− 0.20	1.15	1.61	
	Max temp	0.43	0.29	0.57	1.54	***
	Min temp	− 0.03	− 0.26	0.20	0.97	
umkh	DTR	− 0.10	− 0.45	0.25	0.90	
	Max temp	0.58	0.39	0.76	1.78	***
	Min temp	0.19	− 0.30	0.68	1.21	
umzn	DTR	0.05	0.21	0.38	− 0.383, 0.477	
	Max temp	0.28	0.16	0.41	1.32	***
	Min temp	− 0.09	− 0.29	0.12	0.92	
uthk	DTR	0.35	− 0.03	0.73	1.42	*
	Max temp	0.26	0.13	0.39	1.30	***
	Min temp	0.02	− 0.12	0.17	1.02	
uthn	DTR	0.05	− 0.42	0.52	1.05	
	Max temp	0.29	0.12	0.47	1.34	***
	Min temp	− 0.06	− 0.30	0.18	0.94	
vhmb	DTR	− 1.08	− 1.63	− 0.52	0.34	***
	Max temp	0.56	0.37	0.75	1.75	***
	Min temp	0.15	− 0.12	0.41	1.16	
wstc	DTR	− 0.18	− 0.63	0.27	0.84	
	Max temp	0.25	0.07	0.43	1.28	***
	Min temp	0.13	− 0.14	0.40	1.14	
wstr	DTR	0.08	− 0.37	0.52	1.08	
	Max temp	0.20	0.00	0.39	1.22	**
	Min temp	− 0.02	− 0.22	0.19	0.99	
wtrb	DTR	0.18	− 0.31	0.67	1.20	
	Max temp	0.42	0.18	0.67	1.53	***
	Min temp	0.00	− 0.21	0.22	1.00	
xhrp	DTR	− 0.03	− 0.60	0.55	0.97	
	Max temp	0.54	0.24	0.83	1.71	***
	Min temp	− 0.22	− 0.48	0.04	0.80	*
zlln	DTR	− 0.38	− 1.09	0.33	0.69	
	Max temp	0.35	0.12	0.59	1.42	***
	Min temp	0.31	0.01	0.61	1.36	**

*CI* confidence interval, *IRR* incident rate ratio, *max temp* maximum temperature, *min temp* minimum temperature, *DTR* diurnal temperature range *p < 0.1, **p < 0.05, ***p < 0.01

^#^Only the first lag results are shown

### Threshold regression

**Fig. 1:**
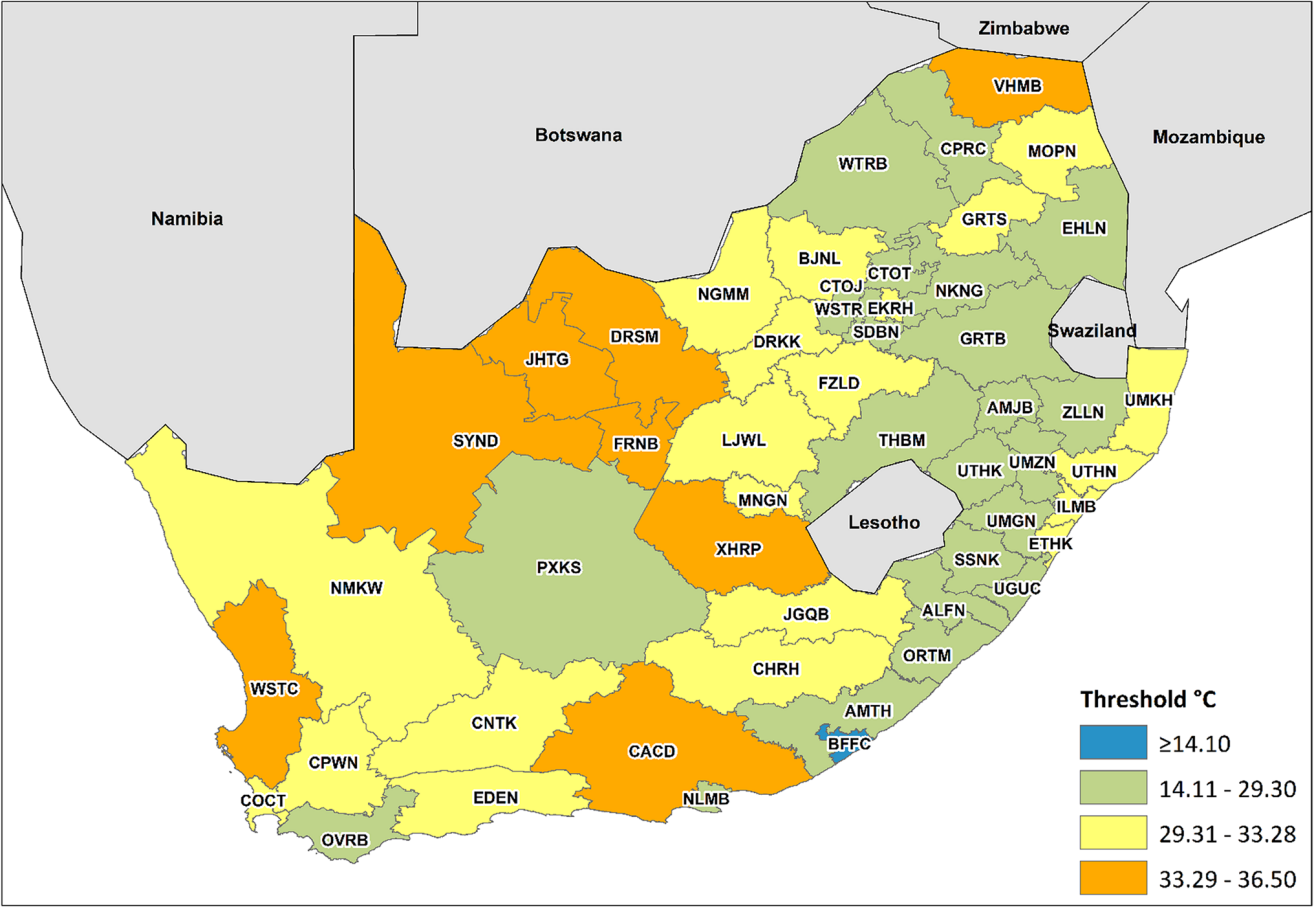
Maximum thresholds (in °C) for mortality for each of the 52 districts in South Africa estimated from threshold regression

**Fig. 2:**
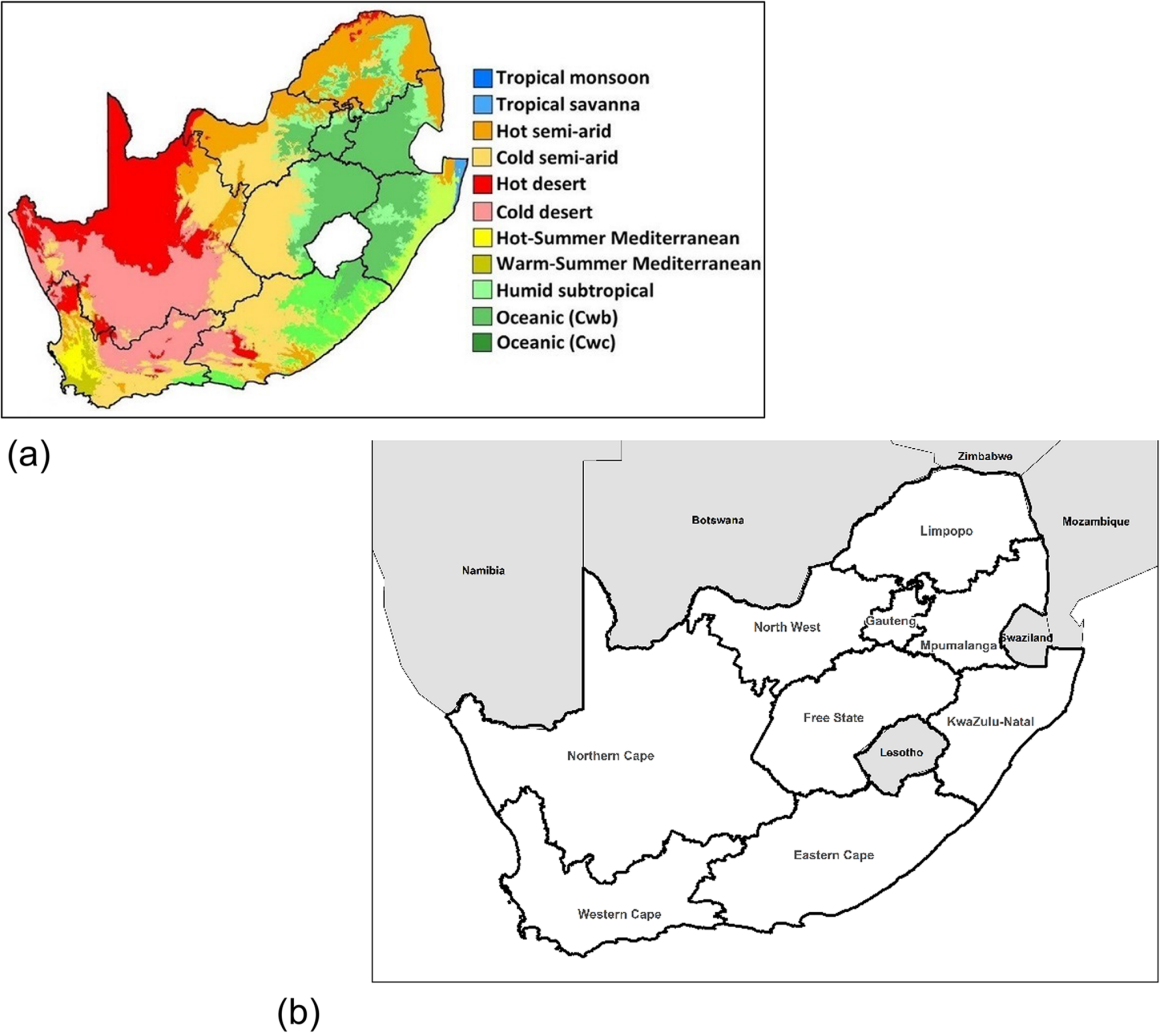
**a** Köppen-Geiger map of the climate zones in South Africa (Beck, Zimmermann et al. 2018). **b** Map depicting provinces of South Africa

Maximum temperature was found to be the most important predictor of mortality. Threshold regression was used to determine district-level threshold values for maximum temperature during the summer months, above which daily mortality increases. The spatial distribution of the thresholds varies according to the climate zones across the country (Fig. 1). It resembles the distribution of climatic regions defined by the Köppen-Geiger classification system (Fig. 2). This is one of the most widely used approaches to classify regions into zones based on temperature and precipitation characteristics. The results of the threshold regression for each district are presented in Table S2. Figure 1 shows that, on average, districts located in the hot arid interior provinces of the Northern Cape and the North West had the highest thresholds (provincial average of 33.31 and 33.05) compared to districts located in the temperate interior (Mpumalanga province) and along the coast (Eastern Cape province) 27.92 and 26.37, respectively. These are among the hottest provinces in the country, and they have experienced several significant heatwaves that have impacted human health in recent years (Marue 2016; Mbokodo et al. 2020; van der Walt and Fitchett 2021).

## Discussion

Our aim was to identify which temperature metric is the most important predictor of mortality during summer months. This metric can then be considered as an exposure outcome towards the development of a HHWS for South Africa. The temperature metrics included in this study were maximum and minimum temperature and DTR. We also calculated district-level thresholds for the most significant metric that can be used to issue heat warnings. An important component of HHWS involves choosing a temperature metric that is most appropriate in terms of prediction for adverse heat-related health outcomes. However, the SAWS definition of extreme heat events does not incorporate the association between an exposure metric and a health outcome. Therefore, our study aimed to provide recommendations based on heat-health evidence.

Based on our analysis of the relationship between three daily temperature metrics and mortality during summer months, maximum temperature was found to be the best predictor of mortality across the country. Daily maximum represents the maximum thermal stress experienced by the human body and is therefore commonly used as an exposure metric in heat-mortality studies (Tan et al. 2007). This has been corroborated by studies that have found strong associations between maximum temperature and mortality (Davis et al. 2016). For example, a study conducted in India, a country with hot summers that has experienced several fatal heatwaves over the years, found high correlations between daily mortality and maximum temperatures during the hottest months of the year (April, May, and June) and all were statistically significant (Azhar et al. 2014). Also, a study in Australia, a region with a climate similar to South Africa, found that maximum temperature had a significant effect on mortality with a 10 °C increase in daily maximum temperature resulting in an increase of 4.5–12.1% in mortality (Vaneckova et al. 2008). In addition, operational HHWS in several countries use the exceedance of a maximum temperature threshold to issue heat alerts (Nogueira 2005; Basarin et al. 2020; NHS 2020; Wu et al. 2020). This supports the feasibility of recommending maximum temperature as a meteorological index for high temperature warnings in South Africa.

We also found spatial variation in the distribution of maximum temperature thresholds related to the climate conditions in local regions. Provinces in hot arid regions experienced high district-level average maximum temperature thresholds. Findings from previous studies suggest that populations living in hotter climates cope better in extreme heat and therefore temperature thresholds for heat-related mortality are higher for warm regions compared to cooler areas (Kenny et al. 2019; Kalkstein et al. 2011). An analysis of daily summer temperatures and mortality across the ten government regions of England and Wales found that regions with hotter climates had higher thresholds for mean temperature compared to colder climates (Armstrong et al. 2011). In a study across seven regions in China, minimum mortality temperature was found to be higher in regions with warmer climates compared to those with cooler climates (Ma et al. 2015). More evidence of the geographical variation of the temperature-mortality relationship was found in a multi-country study where heat thresholds were higher in cities with hotter summers (McMichael et al. 2008).

The findings of our study relating to the high maximum temperature thresholds for hot regions indicate population adaptation to local climate. Previous studies conducted across the world also reported similar observations that support this hypothesis. For example, the national heat index threshold of 40.1 °C used by the US National Weather Service to issue heat alerts was found to be ineffective in the desert communities of California. The threshold was regularly exceeded in these communities, but residents are well adapted to extreme heat (Guirguis et al. 2014). In Croatia, the analysis of mortality and meteorological data over a 26-year period found that thresholds for maximum temperature were higher in continental parts of the country compared to cooler, coastal areas (Zaninović and Matzarakis 2014). Another study that compared temperature and mortality associations in the UK and Australia found that relative risk attributed to the exceedance of heat thresholds was lower in Australian cities than in the UK (Vardoulakis et al. 2014). Some of the suggested reasons were the physiological acclimatization and behavioral adaptation of the population of Australia due to the warmer climate (Vardoulakis et al. 2014).

The World Meteorological Organization and World Health Organization guidance on the development of HHWS acknowledges that there is no preferred meteorological variable or exposure metric that is recommended for use in HHWS (McGregor et al. 2015). Indicators that are used in operational HHWS across the world include maximum temperature, minimum temperature, mean temperature, apparent temperature, and air mass (calculated by combining air temperature, dewpoint temperature, total cloud cover, sea level pressure, windspeed, and wind direction). However, for increased effectiveness, heat indicators in HHWS should be based on variables that are easy to forecast with a certain level of confidence to ensure accurate prediction of heat events. According to Pascal et al. (2006), temperature forecasts up to 5 days in advance are within the acceptance level of confidence for heat early warnings. SAWS can forecast minimum and maximum temperature up to 3 days in advance with a high level of confidence; therefore, our study used maximum and minimum temperature and DTR as input variables. For future research, we recommend that the performance of maximum temperature on forecasted data should also be tested to ensure its effectiveness in a HHWS.

Several limitations were considered during this study. Firstly, we used all-cause mortality and not exposure to excessive natural heat (ICD-10 code X30). Although using heat-specific death would increase reliability of results, heat-related deaths are often misclassified as deaths due to heart attacks, cardiovascular disease, and respiratory disease (Basu and Samet 2002). According to the mortality dataset, there were about 10 deaths per year recorded as being heat-related across South Africa from 1997 to 2013 which suggests gross underreporting (Stats SA 2014). The study also did not account for air pollution (particles, ozone, nitrogen dioxide, sulfur dioxide, and carbon monoxide), rainfall, or humidity which are potential confounder of the temperature–mortality relationship. High temperatures have been associated with increased levels of air pollution which also increases risk of mortality (Hu et al. 2022). Therefore, our results could have potentially overestimated the effect of heat on mortality due to the lack of adjustment for air pollution variables. Lastly, our study used mortality as a health outcome; however, hospitalizations (including emergency department visits) and ambulance callouts have been found to capture heat-related health outcomes more accurately (Bishop-Williams et al. 2015; Li et al. 2015). Unfortunately, these data are difficult to obtain for South Africa due to controlled access to data, the slow pace of migration from hard copy record keeping to computerized records, and challenges with data quality.

## Conclusions

Effective and efficient HHWS require that threshold values be informed by epidemiological studies assessing temperature–mortality relationship. The results of our analysis suggest that the development and implementation of HHWS should be country specific, taking the local climate into account in order to reduce heat-related mortality and morbidity. This study investigated which temperature index (maximum and minimum temperature or a combination of both) has the potential to be incorporated into a HHWS design that takes associations with a health outcome (in this case, mortality) into consideration. Maximum temperature was the most robust predictor of all-cause mortality, and thresholds varied across the country depending on the local climate. Based on the findings, this study recommends a HHWS incorporating district-level maximum temperature thresholds to issue heat alerts.

### Supplementary Information

Below is the link to the electronic supplementary material.

Supplementary file1 (DOCX 58 KB)

## Data Availability

The data that support the findings of this study are available from Statistics South Africa, the National Oceanographic and Atmospheric Administration (NOAA), and South Africa’s Agricultural Research Council (ARC) but restrictions apply to the availability of these data, which were used under license for the current study, and so are not publicly available. Data are however available from the authors upon reasonable request and with permission of data custodians.
