# Supplementary material for: Identifying heat thresholds for South Africa towards the development of a heat-health warning system
Source: Int J Biometeorol. 2023 Dec 29;68(2):381–92. doi: 10.1007/s00484-023-02596-z (PMC10794383; doi:10.1007/s00484-023-02596-z)
Supplement: Supplementary file 1 — Supplementary file1 (DOCX 58 KB) [file 484_2023_2596_MOESM1_ESM.docx]

Table S1: Descriptive statistics for temperature variables and mortality in South Africa for October, November, December, January, February, and March from 1997 to 2013

| **Number** | **District abbreviation** | DC_NAME | PR_NAME | **Total mortality** | **Maximum temperature °C** | **Minimum temperature °C** | **DTR °C** |
| --- | --- | --- | --- | --- | --- | --- | --- |
| 1 | alfn | Alfred Nzo | Eastern Cape | 56281 | 22.86 (8.8 – 37) | 13.51 (3 – 24.5) | 10.1 (7.7 - 13.4) |
| 2 | amjb | Amajuba | KwaZulu-Natal | 45126 | 28.04 (12.36 – 37.72) | 14.81 (2.67 – 20.84) | 15.0 (10.3 - 19.4) |
| 3 | amth | Amathole | Eastern Cape | 115620 | 27.10 (11 – 42.2) | 11.09 (-3.5 – 28.0) | 13.6 (8.8 - 18.8) |
| 4 | bffc | Buffalo City | Eastern Cape | 89256 | 17.69 (7.4 – 26.4) | 10.30 (-0.5 – 20.4) | 6.8 (4.6 - 9.0) |
| 5 | bjnl | Bojanala | North West | 102978 | 29.10 (10.6 – 38.2) | 15.55 (5 – 22.5) | 15.3 (9.4 - 20.2) |
| 6 | cacd | Cacadu | Eastern Cape | 35723 | 28.63 (11.5 – 45.0) | 14.89 (1.8 – 27.8) | 14.0 (8.5 - 18.1) |
| 7 | chrh | Chris Hani | Eastern Cape | 68750 | 27.40 (8.0 – 41.3) | 11.77 (-0.9 – 23.5) | 15.6 (11.1 - 19.4) |
| 8 | cntk | Central Karoo | Western Cape | 6547 | 29.96 (13.4 – 43.5) | 14.66 (1 – 27.2) | 13.9 (9.1 - 18.6) |
| 9 | coct | City of Cape Town | Western Cape | 208147 | 26.70 (14.8 – 41.3) | 14.71 (0 – 23.5) | 12.0 (8.7 - 14.7) |
| 10 | cprc | Capricorn | Limpopo | 98566 | 29.0 (13.7 – 37.8) | 15.72 (5.3 – 23.4) | 15.0 (8.8 - 20.2) |
| 11 | cpwn | Cape Winelands | Western Cape | 47780 | 27.16 (8.5 – 39.0) | 12.04 (-1.0 – 24.5) | 14.0 (9.1 - 17.0) |
| 12 | ctoj | City of Johannesburg | Gauteng | 255127 | 25.88 (11.2 – 34.1) | 13.07 (-2.4 – 19.8) | 13.5 (9.5 - 17.4) |
| 13 | ctot | City of Tshwane | Gauteng | 173343 | 26.76 (8.0 – 35.1) | 13.44 (-1.0 – 19.6) | 16.6 (8.8 - 26.2) |
| 14 | drkk | Dr Kenneth Kaunda | North West | 73742 | 30.34 (14.3 – 41.3) | 14.65 (0.5 – 23.5) | 18.2 (11.24 - 23.9) |
| 15 | drsm | Dr Ruth Segomotsi Mompati | North West | 41046 | 33.10 (17.9 – 42.5) | 16.18 (1.5 – 24.9) | 18.3 (11.4 - 23.9) |
| 16 | eden | Eden | Western Cape | 38943 | 28.08 (7.8 – 43.0) | 13.82 (0 – 27.4) | 13.2 (9.4 - 16.9) |
| 17 | ehln | Ehlanzeni | Mpumalanga | 126256 | 28.75 (14.6 – 42.0) | 17.57 (7 - 23.2) | 13.5 (9.0 - 20.4) |
| 18 | ekrh | Ekurhuleni | Gauteng | 207478 | 26.0 (6.5 – 34.7) | 13.09 (1.5 – 18.6) | 14.3 (9.5 - 18.3) |
| 19 | ethk | eThekwini | KwaZulu-Natal | 284598 | 28.0 (17.5 – 39.5) | 19.49 (8.0 - 25.5) | 10.2 (6.9 - 16.3) |
| 20 | frnb | Frances Baard | Northern Cape | 38435 | 31.9 (14.8 – 40.9) | 14.96 (-0.7 – 24.7) | 17.6 (11.6 - 23.0) |
| 21 | fzld | Fezile Dabi | Free State | 50283 | 28.5 (12.2 – 37.3) | 13.43 (-2.3 – 19.7) | 17.4 (10.1 - 23.2) |
| 22 | grtb | Gert Sibande | Mpumalanga | 96696 | 24.6 (8.4 – 34.6) | 13.05 (1.6 – 19.6) | 12.9 (7.8 - 17.2) |
| 23 | grts | Greater Sekhukhune | Limpopo | 74587 | 31.1 (17.4 – 45.2) | 17.37 (6.53 – 24.5) | 16.1 (10.4 - 21.2) |
| 24 | ilmb | iLembe | KwaZulu-Natal | 48231 | 27.7 (16.0 – 43.5) | 18.67 (11.4 – 25.7) | 10.1 (7.6 - 13.5) |
| 25 | jgqb | Joe Gqabi | Eastern Cape | 34335 | 28.7 (12.9 – 38.8) | 11.18 (-2.4 – 19.7) | 17.7 (11.6 - 22.1) |
| 26 | jhtg | John Taolo Gaetsewe | Northern Cape | 18461 | 32.1 (12.7 – 40.6) | 14.82 (1.65 – 24.97) | 18.5 (12.8 - 23.5) |
| 27 | ljwl | Lejweleputswa | Free State | 82809 | 29.4 (12.7 – 45.2) | 13.32 (-2.0 – 21.7) | 18.0 (9.0 - 29.3) |
| 28 | mngn | Mangaung | Free State | 87439 | 30.1 (16.3 – 39.7) | 12.38 (-1.8 – 21.7) | 19.0 (10.7 - 25.0) |
| 29 | mopn | Mopani | Limpopo | 74904 | 31.0 (16.3 – 42.7) | 18.75 (7.2 – 27.7) | 14.5 (8.8 - 20.5) |
| 30 | ngmm | Ngaka Modiri Molema | North West | 74980 | 30.6 (14.2 – 39.2) | 16.42 (5.5 – 23.7) | 15.4 (9.9 - 20.0) |
| 31 | nkng | Nkangala | Mpumalanga | 92517 | 27.2 (11.9 – 35.5) | 12.44 (-0.3 – 17.67) | 17.6 (10.7 - 24.1) |
| 32 | nlmb | Nelson Mandela Bay | Eastern Cape | 102065 | 25.2 (15.8 – 39.6) | 14.66 (2.2 – 23) | 12.2 (8.1 - 18.1) |
| 33 | nmkw | Namakwa | Northern Cape | 7595 | 29.5 (9.4 – 39.4) | 14.69 (-0.8 – 27.2) | 13.3 (8.6 - 17.6) |
| 34 | ortm | O.R.Tambo | Eastern Cape | 96968 | 25.9 (11.1 – 39.9) | 14.29 (2.7 – 22.2) | 13.6 (8.7 - 19.7) |
| 35 | ovrb | Overberg | Western Cape | 14494 | 26.5 (12.9 – 42.5) | 13.48 (-0.6 – 23.2) | 13.1 (7.8 - 16.8) |
| 36 | pxks | Pixley ka Seme | Northern Cape | 19739 | 31.1 (14.8 – 39.9) | 14.16 (0.2 – 23.6) | 16.3 (12.02 - 19.8) |
| 37 | sdbn | Sedibeng | Gauteng | 80709 | 28.1 (12.4 – 36.0) | 14.10 (0.5 – 20.3) | 16.3 (10.0 - 23.1) |
| 38 | ssnk | Sisonke | KwaZulu-Natal | 45263 | 23.3 (6.0 – 33.0) | 10.79 (-2.5 – 21.7) | 14.5 (8.3 - 20.5) |
| 39 | synd | Siyanda | Northern Cape | 22560 | 34.6 (13.8 – 44.0) | 16.70 (1.6 – 28.0) | 18.5 (12.9 - 23.1) |
| 40 | thbm | Thabo Mofutsanyane | Free State | 94459 | 26.4 (12.5 – 36.4) | 11.26 (-2.9 – 18.3) | 16.9 (110 - 22.2) |
| 41 | uguc | Ugu | KwaZulu-Natal | 83497 | 26.1 (15.4 – 35.7) | 19.42 (10.7 – 25.5) | 7.5 (5.1 - 9.9) |
| 42 | umgn | UMgungundlovu | KwaZulu-Natal | 109933 | 21.6 (5.3 – 33.5) | 11.10 (-0.8 – 20.8) | 11.6 (51 - 16.4) |
| 43 | umkh | Umkhanyakude | KwaZulu-Natal | 45246 | 29.4 (17.7 – 47.3) | 19.93 (-12.6 – 26.6) | 10.6 (7.5 - 13.5) |
| 44 | umzn | Umzinyathi | KwaZulu-Natal | 51688 | 24.5 (9.5 – 37.7) | 13.50 (0.6 – 24.4) | 13.3 (8.1 - 20.3) |
| 45 | uthk | Uthukela | KwaZulu-Natal | 68004 | 25.1 (9.6 – 34.1) | 12.30 (-0.3 – 19.1) | 14.5 (9.4 - 20.0) |
| 46 | uthn | Uthungulu | KwaZulu-Natal | 89413 | 31.0 (16.7 – 42.8) | 19.25 (4.7 – 26.9) | 13.1 (9.5 - 19.2) |
| 47 | vhmb | Vhembe | Limpopo | 65985 | 33.4 (18.9 – 43.8) | 20.24 (5.66 – 27.8) | 16.5 (9.0 - 21.9) |
| 48 | wstc | West Coast | Western Cape | 27636 | 30.7 (14.0 – 44.7) | 14.92 (2.7 – 27.9) | 14.2 (9.0 - 18.1) |
| 49 | wstr | West Rand | Gauteng | 74630 | 26.6 (11.2 – 34.7) | 14.14 (1.9 – 19.4) | 13.9 (9.1 - 18.2) |
| 50 | wtrb | Waterberg | Limpopo | 35866 | 31.8 (14.0 – 47.1) | 19.18 (7.53 – 27.11) | 15.0 (9.1 - 21.4) |
| 51 | xhrp | Xhariep | Free State | 23898 | 31.5 (12.1 – 46.8) | 13.52 (-0.6 – 24.9) | 18.7 (12.8 - 28.9) |
| 52 | zlln | Zululand | KwaZulu-Natal | 57644 | 28.0 (10.0 – 39.4) | 14.55 (-0.5 – 22.3) | 15.0 (9.4 - 20.0) |

Table S2: Maximum temperature thresholds for districts in South Africa

| **District** | **Threshold °C** | **Region** | **Model coefficient** | **P-value** | **95% Confidence Interval** | |
| --- | --- | --- | --- | --- | --- | --- |
| alfn | 21.99 | Region 1 | 0.23 | <0.001 | 0.23 | 0.24 |
|  |  | Region 2 | 0.25 | <0.001 | 0.25 | 0.26 |
| amjb | 27.79 | Region 1 | 0.32 | <0.001 | 0.31 | 0.33 |
|  |  | Region 2 | 0.34 | <0.001 | 0.33 | 0.35 |
| amth | 25.90 | Region 1 | 0.39 | <0.001 | 0.38 | 0.40 |
|  |  | Region 2 | 0.43 | <0.001 | 0.42 | 0.43 |
| bffc | 14.10 | Region 1 | 0.23 | <0.001 | 0.22 | 0.24 |
|  |  | Region 2 | 0.22 | <0.001 | 0.21 | 0.22 |
| bjnl | 33.28 | Region 1 | 0.43 | <0.001 | 0.43 | 0.44 |
|  |  | Region 2 | 0.50 | <0.001 | 0.49 | 0.51 |
| cacd | 34.80 | Region 1 | 0.26 | <0.001 | 0.26 | 0.27 |
|  |  | Region 2 | 0.30 | <0.001 | 0.29 | 0.30 |
| chrh | 31.30 | Region 1 | 0.30 | <0.001 | 0.30 | 0.31 |
|  |  | Region 2 | 0.33 | <0.001 | 0.32 | 0.34 |
| cntk | 32.90 | Region 1 | 0.02 | <0.001 | 0.02 | 0.02 |
|  |  | Region 2 | 0.02 | <0.001 | 0.02 | 0.02 |
| coct | 31.00 | Region 1 | 9.57 | <0.001 | 9.50 | 9.64 |
|  |  | Region 2 | 10.04 | <0.001 | 9.87 | 10.21 |
| cprc | 29.10 | Region 1 | 0.35 | <0.001 | 0.35 | 0.36 |
|  |  | Region 2 | 0.38 | <0.001 | 0.38 | 0.39 |
| cpwn | 30.00 | Region 1 | 0.04 | <0.001 | 0.04 | 0.04 |
|  |  | Region 2 | 0.05 | <0.001 | 0.05 | 0.05 |
| ctoj | 29.30 | Region 1 | 0.22 | <0.001 | 0.22 | 0.22 |
|  |  | Region 2 | 0.24 | <0.001 | 0.23 | 0.24 |
| ctot | 29.00 | Region 1 | 0.22 | <0.001 | 0.22 | 0.22 |
|  |  | Region 2 | 0.24 | <0.001 | 0.24 | 0.24 |
| drkk | 31.40 | Region 1 | 0.37 | <0.001 | 0.37 | 0.38 |
|  |  | Region 2 | 0.36 | <0.001 | 0.36 | 0.37 |
| drsm | 35.05 | Region 1 | 0.29 | <0.001 | 0.28 | 0.29 |
|  |  | Region 2 | 0.33 | <0.001 | 0.32 | 0.33 |
| eden | 31.80 | Region 1 | 0.23 | <0.001 | 0.22 | 0.23 |
|  |  | Region 2 | 0.25 | <0.001 | 0.24 | 0.25 |
| ehln | 28.67 | Region 1 | 0.26 | <0.001 | 0.25 | 0.26 |
|  |  | Region 2 | 0.29 | <0.001 | 0.28 | 0.29 |
| ekrh | 29.57 | Region 1 | 0.25 | <0.001 | 0.25 | 0.25 |
|  |  | Region 2 | 0.27 | <0.001 | 0.26 | 0.28 |
| ethk | 30.60 | Region 1 | 0.29 | <0.001 | 0.28 | 0.29 |
|  |  | Region 2 | 0.28 | <0.001 | 0.27 | 0.28 |
| frnb | 35.30 | Region 1 | 0.25 | <0.001 | 0.24 | 0.25 |
|  |  | Region 2 | 0.27 | <0.001 | 0.27 | 0.28 |
| fzld | 31.48 | Region 1 | 0.46 | <0.001 | 0.45 | 0.47 |
|  |  | Region 2 | 0.49 | <0.001 | 0.32 | 0.33 |
| grtb | 26.29 | Region 1 | 0.33 | <0.001 | 0.32 | 0.33 |
|  |  | Region 2 | 0.35 | <0.001 | 0.35 | 0.36 |
| grts | 30.97 | Region 1 | 0.25 | <0.001 | 0.25 | 0.25 |
|  |  | Region 2 | 0.27 | <0.001 | 0.26 | 0.27 |
| ilmb | 31.78 | Region 1 | 0.28 | <0.001 | 0.28 | 0.29 |
|  |  | Region 2 | 0.33 | <0.001 | 0.32 | 0.34 |
| jgqb | 30.79 | Region 1 | 0.36 | <0.001 | 0.36 | 0.37 |
|  |  | Region 2 | 0.39 | <0.001 | 0.38 | 0.40 |
| jhtg | 34.56 | Region 1 | 0.31 | <0.001 | 0.30 | 0.32 |
|  |  | Region 2 | 0.34 | <0.001 | 0.32 | 0.35 |
| ljwl | 32.86 | Region 1 | 0.43 | <0.001 | 0.43 | 0.44 |
|  |  | Region 2 | 0.45 | <0.001 | 0.44 | 0.46 |
| mngn | 32.10 | Region 1 | 0.37 | <0.001 | 0.36 | 0.37 |
|  |  | Region 2 | 0.39 | <0.001 | 0.38 | 0.39 |
| mopn | 32.68 | Region 1 | 0.24 | <0.001 | 0.24 | 0.24 |
|  |  | Region 2 | 0.26 | <0.001 | 0.26 | 0.27 |
| ngmm | 32.50 | Region 1 | 2.04 | <0.001 | 2.01 | 2.06 |
|  |  | Region 2 | 2.20 | <0.001 | 2.16 | 2.25 |
| nkng | 28.79 | Region 1 | 0.29 | <0.001 | 0.29 | 0.29 |
|  |  | Region 2 | 0.30 | <0.001 | 0.30 | 0.31 |
| nlmb | 25.10 | Region 1 | 0.39 | <0.001 | 0.39 | 0.40 |
|  |  | Region 2 | 0.43 | <0.001 | 0.42 | 0.43 |
| nmkw | 32.90 | Region 1 | 0.02 | <0.001 | 0.02 | 0.02 |
|  |  | Region 2 | 0.02 | <0.001 | 0.02 | 0.02 |
| ortm | 27.00 | Region 1 | 0.23 | <0.001 | 0.23 | 0.23 |
|  |  | Region 2 | 0.25 | <0.001 | 0.24 | 0.25 |
| ovrb | 28.10 | Region 1 | 0.20 | <0.001 | 0.20 | 0.21 |
|  |  | Region 2 | 0.21 | <0.001 | 0.20 | 0.22 |
| pxks | 29.10 | Region 1 | 0.33 | <0.001 | 3177877.00 | 0.34 |
|  |  | Region 2 | 0.36 | <0.001 | 0.35 | 0.37 |
| sdbn | 28.95 | Region 1 | 0.31 | <0.001 | 0.30 | 0.31 |
|  |  | Region 2 | 0.33 | <0.001 | 0.32 | 0.33 |
| ssnk | 26.50 | Region 1 | 0.31 | <0.001 | 0.30 | 0.31 |
|  |  | Region 2 | 0.35 | <0.001 | 0.34 | 0.36 |
| synd | 34.70 | Region 1 | 0.31 | <0.001 | 0.30 | 0.31 |
|  |  | Region 2 | 0.34 | <0.001 | 0.34 | 0.35 |
| thbm | 28.30 | Region 1 | 0.40 | <0.001 | 0.39 | 0.40 |
|  |  | Region 2 | 0.42 | <0.001 | 0.41 | 0.43 |
| uguc | 28.24 | Region 1 | 0.40 | <0.001 | 0.40 | 0.41 |
|  |  | Region 2 | 0.41 | <0.001 | 0.40 | 0.42 |
| umgn | 23.70 | Region 1 | 0.35 | <0.001 | 0.34 | 0.35 |
|  |  | Region 2 | 0.39 | <0.001 | 0.39 | 0.40 |
| umkh | 30.89 | Region 1 | 0.29 | <0.001 | 0.29 | 0.30 |
|  |  | Region 2 | 0.33 | <0.001 | 0.32 | 0.34 |
| umzn | 26.20 | Region 1 | 0.27 | <0.001 | 0.26 | 0.27 |
|  |  | Region 2 | 0.30 | <0.001 | 0.30 | 0.31 |
| uthk | 24.60 | Region 1 | 0.32 | <0.001 | 0.32 | 0.33 |
|  |  | Region 2 | 0.35 | <0.001 | 0.34 | 0.35 |
| uthn | 33.16 | Region 1 | 0.32 | <0.001 | 0.32 | 0.33 |
|  |  | Region 2 | 0.34 | <0.001 | 0.34 | 0.35 |
| vhmb | 36.31 | Region 1 | 0.19 | <0.001 | 0.19 | 0.19 |
|  |  | Region 2 | 0.21 | <0.001 | 0.21 | 0.22 |
| wstc | 36.50 | Region 1 | 0.14 | <0.001 | 0.13 | 0.14 |
|  |  | Region 2 | 0.15 | <0.001 | 0.15 | 0.16 |
| wstr | 26.29 | Region 1 | 0.70 | <0.001 | 0.69 | 0.71 |
|  |  | Region 2 | 0.75 | <0.001 | 0.74 | 0.77 |
| wtrb | 27.26 | Region 1 | 0.14 | <0.001 | 0.14 | 0.15 |
|  |  | Region 2 | 0.16 | <0.001 | 0.16 | 0.16 |
| xhrp | 33.74 | Region 1 | 0.61 | <0.001 | 0.60 | 0.63 |
|  |  | Region 2 | 0.66 | <0.001 | 0.65 | 0.68 |
| zlln | 28.07 | Region 1 | 0.24 | <0.001 | 0.24 | 0.24 |
|  |  | Region 2 | 0.25 | <0.001 | 0.25 | 0.25 |
